# Improving the regeneration rate of deep lethal mutant protoplasts by fusion to promote efficient L-lysine fermentation

**DOI:** 10.1186/s12896-023-00792-8

**Published:** 2023-07-14

**Authors:** Nan Li, Jie Lu, Zirui Wang, Peng Du, Piwu Li, Jing Su, Jing Xiao, Min Wang, Junqing Wang, Ruiming Wang

**Affiliations:** 1 College of Biotechnology, Tianjin University of Science and Technology, Tianjin, 300457 China; 2 State Key Laboratory of Biobased Material and Green Papermaking (LBMP), Qilu University of Technology, Jinan, 250353 China

**Keywords:** L-lysine, *Escherichia coli*, Protoplasts, Mutation breeding, Homologous cell fusion

## Abstract

**Background:**

L-lysine is widely used for feed and special diet products. The transformation of fermentation strains plays a decisive role in the development of these industries. Based on the mutation breeding theory and metabolic engineering methods, this study aimed to improve the regeneration rate of high-lethality protoplasts by combining multiple mutagenesis and homologous cell fusion techniques to efficiently concentrate multiple dominant mutations and optimize the L-lysine production strain *Escherichia coli* QDW.

**Results:**

In order to obtain the best protoplasts, the optimal enzymolysis time was selected as 4 h. The optimal lysozyme concentration was estimated at 0.8 mg/mL, because the protoplast formation rate and regeneration rate reached 90% and 30%, respectively, and their product reached the maximum. In this study, it was necessary that UV mutagenesis be excessive to obtain an expanded mutation library. For high lethality protoplasts, under the premise of minimal influence on its recovery, the optimal time for UV mutagenesis of protoplasts was 7 min, and the optimal time for thermal inactivation of protoplasts at 85 ℃ was 30 min. After homologous fusion, four fusion strains of *E. coli* were obtained, and their stability was analyzed by flow cytometry. The L-lysine yield of QDW-UH3 increased by 7.2% compared with that of QDW in a fermentation experiment, which promoted the expression of key enzymes in L-lysine synthesis, indicating that the combination of ultraviolet mutagenic breeding and protoplast fusion technology improved the acid-production level of the fusion strain.

**Conclusion:**

This method provides a novel approach for the targeted construction of microbial cell factories.

**Supplementary Information:**

The online version contains supplementary material available at 10.1186/s12896-023-00792-8.

## Background

L-lysine is widely used in farmed feed, with 90% of L-lysine used as feed additives in animal husbandry. Adding L-lysine to ordinary feed remarkably improves the utilization rate of the feed [1–3]. Intake of L-lysine during the growth stage can promote the growth of domestic animals and poultry and reduce raw material costs. L-lysine can be used as an auxiliary agent of diuretic drugs [4] and is used primarily as a food fortification agent and food deodorant in the food industry [5–7]. In recent years, scientists have adopted various methods to modify *Escherichia coli* and *Corynebacterium glutamicum* to improve L-lysine production. The transformation of L-lysine-producing strains is mainly achieved through processes such as mutagenic breeding, physical and chemical methods to mutate the genes of strains, screening and breeding of nutrition-deficient strains, overexpression of key enzyme genes related to L-lysine synthesis, expansion of substrate range, improvement of carbon flux, as well as accumulation of L-lysine in metabolic pathways [8, 9]. With the continuous development of the feed and breeding industries, the problems facing these industries include stricter requirements for feed safety, increased risk of major diseases, increasing pressure of environmental pollution, lack of feed resources, and rising production costs, which are related to the sustainable development of the feed industry.

The microbial mutagenesis breeding technology has several advantages, including simple operation, high mutagenesis rate, and wide application through comparative analysis of genome sequencing to verify gene mutations, base replacement, and deletion. Radiation mutagenesis, such as that using alpha rays, X rays, neutron particles, ultraviolet radiation, and microwave radiation, is now widely used [10]. For example, Yu et al. applied genome shuffling to improve L-lactic acid production. The starting population was generated by ultraviolet irradiation and nitrosoguanidine mutagenesis and then subjected to recursive protoplast fusion [11]. Furthermore, Xu et al. reported that genome shuffling improves the production of pristinamycin by enhancing product resistance in the spores of *Streptomyces pristinaespiralis* CGMCC0957 [12]. The development of the mutagenesis breeding technology plays an important role in developing microbial drugs [13].

The microbial protoplast fusion technology comprises four steps: protoplast preparation, protoplast fusion, protoplast regeneration, and fusion screening [14, 15]. In order to obtain more excellent characters and gene phenotypes, protoplasts are treated with various mutation methods to obtain dominant mutations; however, the lethality of such methods is high and the mutated cells are difficult to regenerate. Protoplast preparation comprises the removal of cell walls, mainly using enzymes (most commonly lysozyme) [16–19]. Protoplast fusion can be induced using various chemical, physical, and biological methods. The fusion methods that have been widely adopted and confirmed are the polyethylene glycol (PEG) method, high calcium and high pH method, and electrofusion method. Protoplast regeneration is affected by many factors, such as bacterial age, lysozyme concentration, and enzymatic hydrolysis time [20]. Many screening methods exist for protoplast fusion breeding; these mainly involve genetic markers to select fusion strains. There are various types of markers for fusion screening: (1) The trophic type is used as a genetic marker, and the fusion microorganism exhibits two different nutritional deficiencies of both parents [21]; (2) Antibiotics could be used as genetic markers [22]; (3) Inactivated protoplasts are used as genetic markers; fusion microorganisms cannot regenerate independently under different inactivated conditions but can regenerate only through lethal damage complementary protoplast fusion; (4) Fluorescent staining can be used as a genetic marker [23]; (5) The carbon source could serve as a genetic marker; (6) The difference in raw materials could act as a genetic marker [24, 25]; (7) Some special physiological characteristics could be genetic markers. Ferenczy et al. used centrifugal force induction to induce protoplast fusion of nutrient-deficient mutant strains [26]. Hopwood et al. proposed that protoplast fusion and recombination might lead to the expression of some recessive genes or random generation of new gene phenotypes, thus suggesting a new approach for breeding antibiotic-producing bacteria [27]. In the process of protoplasmic preparation, some studies have used mixed enzyme solutions to improve the effect of removing walls [28]. Within a certain range, the time of enzyme action and concentration of enzyme are positively correlated with the protoplast formation rate and inversely correlated with the regeneration rate [29]. In a previous study, EDTA was added after enzymatic hydrolysis to alter the ionic strength and osmotic pressure in the enzymatic hydrolysis environment and improve the protoplast formation rate [30]. Different microorganisms require different enzymes (as well as different enzyme amounts and types) when preparing protoplasts. Consequently, different measurements should be made, based on the various microorganisms used [31–33]. As one of the traditional methods of protoplast fusion breeding, improving the rate of protoplast regeneration to achieve stable growth of fusion strains under the premise of high mutation lethality is essential [34].

Screening of L-lysine fermentation strains and studies related to mutagenesis breeding showed that *Bacillus subtilis* was used as the starting strain, MNNG mutagenesis was treated, AEC plate was used for screening of anti-feedback inhibition of aspartic kinase mutant, and UV (ultraviolet) combined mutagenesis was used to obtain the mutant with acid production up to 21 g/L [35, 36]. Using *Breubacter xanthosus* FM84-415 as the starting strain, the threonine and homoserine dual nutrient deficiency type was obtained by nitroso guanidine mutagination. Meanwhile, the mutant resistant to AEC and MT was found to produce 64.3 g/L acid. Using *Saccharomyces cerevisiae* as the starting strain, adding lysine structural analogue AEC and using proline as the only nitrogen source, the acid yield of the mutant was 3.7 times that of the starting strain [37]. Using AL039 as the starting strain, a fluoropyruvate sensitive mutant was screened by nitroso guanidine mutagenic mutagination, and its acid yield was 40.3 g/L [38].

In this study, we aimed to identify homologous fusion conditions which lead to improvements in the protoplast regeneration rate despite high mutation lethality, such that the dominant mutant strain can grow stably. We identified the optimal bacterial age, lysozyme concentration, enzymatic hydrolysis time, heat inactivation time, and ultraviolet mutagenesis time for protoplast preparation for the L-lysine producing strain *E. coli* QDW. The dominant mutation was identified by screening with high lethality under extreme conditions, and the regeneration rate of the bacteria was improved under appropriate fusion conditions. The fusion strains *E. coli* QDW-UH1, *E. coli* QDW-UH2, *E. coli* QDW-UH3, and *E. coli* QDW-UH4 were obtained by combining the protoplast fusion technology with ultraviolet mutation breeding technology. The regenerated fusion strains were analyzed and screened by flow cytometry, followed by stability analysis and fermentation experiments. An engineered *E. coli* QDW-UH3 with higher L-lysine production was obtained. Excessive ultraviolet mutagenesis would be expected to cause the accumulation of mutations and yield more positive mutations, but with the consequence of challenges relating to stabilizing growth inheritance. Therefore, we introduced homologous cell fusion, which promoted the expression of key enzymes and increased the accumulation of target products. This efficient and concentrated dominant mutation method provides a novel approach for the targeted construction of microbial cell factories.

## Results

### Optimization of preparation conditions for E. coli QDW protoplasm

**Fig. 1:**
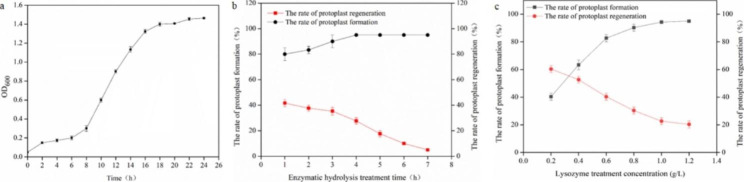
The selection of optimal preparation conditions for *Escherichia coli* QDW protoplasts. (**a**) The growth curve of L-lysine-producing strain QDW. (**b**) The effect of enzymatic hydrolysis time on protoplast formation rate and regeneration rate. (c) The effect of lysozyme concentration on protoplast formation and regeneration rate

There are four periods in bacterial growth: lag, logarithmic, stationary, and death. The length of these four periods changes with different inoculation amounts and culture conditions. The easiest periods for preparation of protoplasts are the mid and late stages of the logarithmic growth phase. The content of peptidoglycan in the bacterial cell wall at the mid and late stages of logarithmic growth is the lowest. Protoplasts with the most significant quantity can be obtained by adding lysozyme to prepare protoplasts in this period. Therefore, the protoplasts were prepared by selecting QDW at the mid and late stages of logarithmic growth. According to Fig. 1a, the mid and late logarithmic growth stage of this strain was between 14 and 16 h. The protoplasts were diluted with SMM buffer solution and uniformly coated on LB regeneration medium, and cultured in an incubator at 37℃. The regeneration rate was determined by the plate regeneration colony. When enzymolysis was conducted for 4 h, the value multiplied by protoplast formation rate and protoplast regeneration rate is the largest. The protoplast formation rate of the original strain was 95%, and the protoplast regeneration rate was 32%. Therefore, the optimal enzymolysis time was selected as 4 h (Fig. 1b). The optimal lysozyme concentration was estimated at 0.8 mg/mL, because the protoplast formation rate and regeneration rate reached 90% and 30%, respectively, and their product reached the maximum (Fig. 1c).

### Determination of UV inactivation time and thermal inactivation time

In this experiment, it was found that the length of inactivation time played a crucial role in the subsequent protoplast fusion rate during the inactivation of protoplasts. If the inactivation time was short, the protoplasm that has not been inactivated will regenerate, which increased the difficulty of fusion screening. If the inactivation time is too long, it may cause irreparable damage to the protoplasts of the parent strains and permanently inactivate them, leading to a decrease in the fusion rate.

**Fig. 2:**
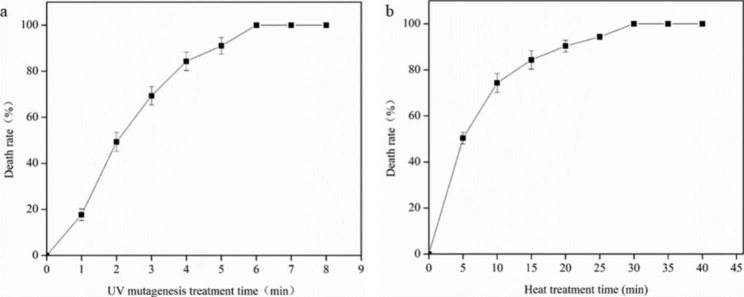
Selection of *Escherichia coli* QDW protoplast inactivation conditions. (**a**) UV mutagenesis death curve. (**b**) Thermal inactivation death curve

The UV inactivation rate reached 100% when the UV irradiation time exceeded 6 min, and the thermal inactivation rate reached 100% when the thermal inactivation time exceeded 30 min (Fig. 2a and b).

In this study, it was necessary that UV mutagenesis be excessive to obtain an expanded mutation library. Under the premise of minimal influence on protoplast recovery, the optimal time for UV mutagenesis of protoplasts was 7 min, and the optimal time for thermal inactivation of protoplasts at 85 ℃ was 30 min (Table 1).

**Table 1:** Parent fusion growth after inactivation

85 ℃ inactivation time UV inactivation time	30 min	35 min	40 min
6 min	√	×	×
7 min	√	×	×
8 min	×	×	×

### Fusion of protoplasts

**Fig. 3:**
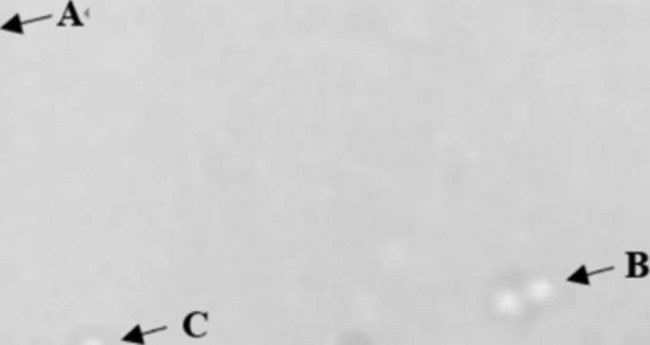
State diagram of protoplast fusion. “**a**” indicates a single protoplast with complete enzymatic hydrolysis, “**b**” indicates the fusion of two protoplasts, and “c” indicates the completed protoplast

The fusion process was observed under an optical microscope, and the strain morphology was recorded at each stage during preparation of the protoplasm (Fig. 3). A is a single protoplast with complete enzymatic hydrolysis, B is a fusion of two protoplasts, and C is a protoplast with complete fusion.

### High-throughput screening by flow cytometry

**Fig. 4:**
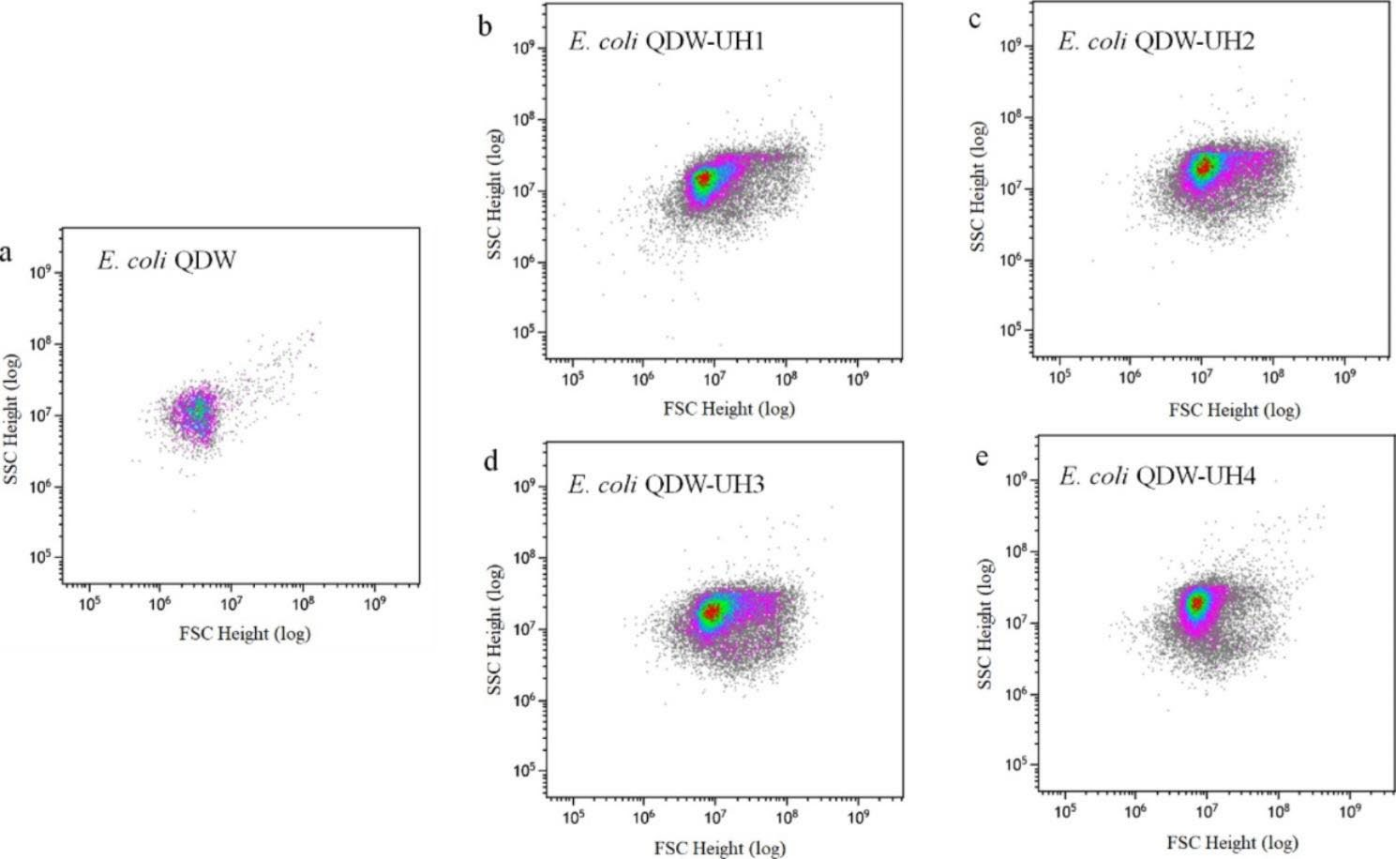
Comparison of size and content of inclusions between QDW and fusion strains

**Fig. 5:**
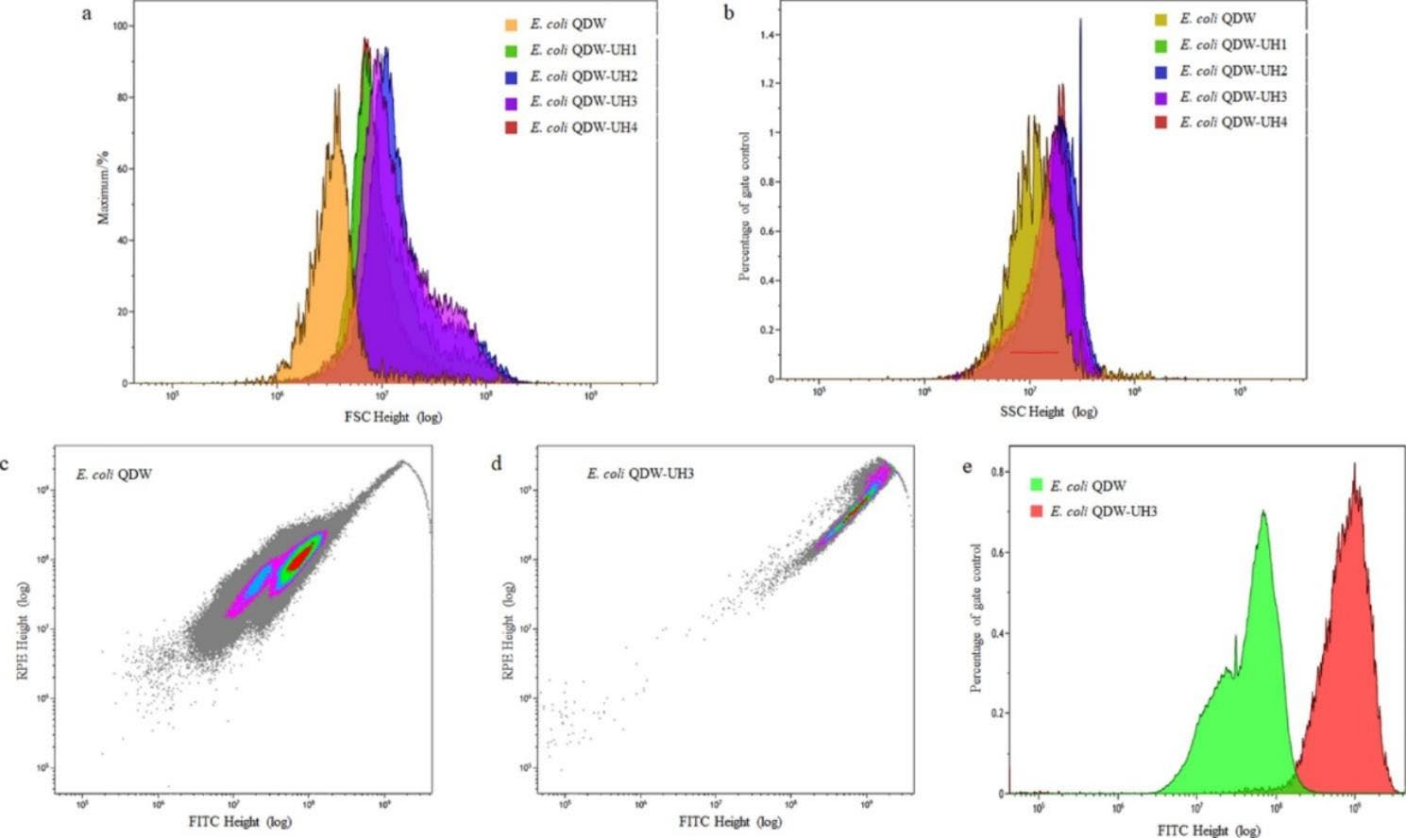
Flow cytometry plots of QDW and fusion strains. (**a**) Comparison of volume size. (**b**) Comparison of contents. (**c**)-(**e**) Comparison plot of genome size

**Fig. 6:**
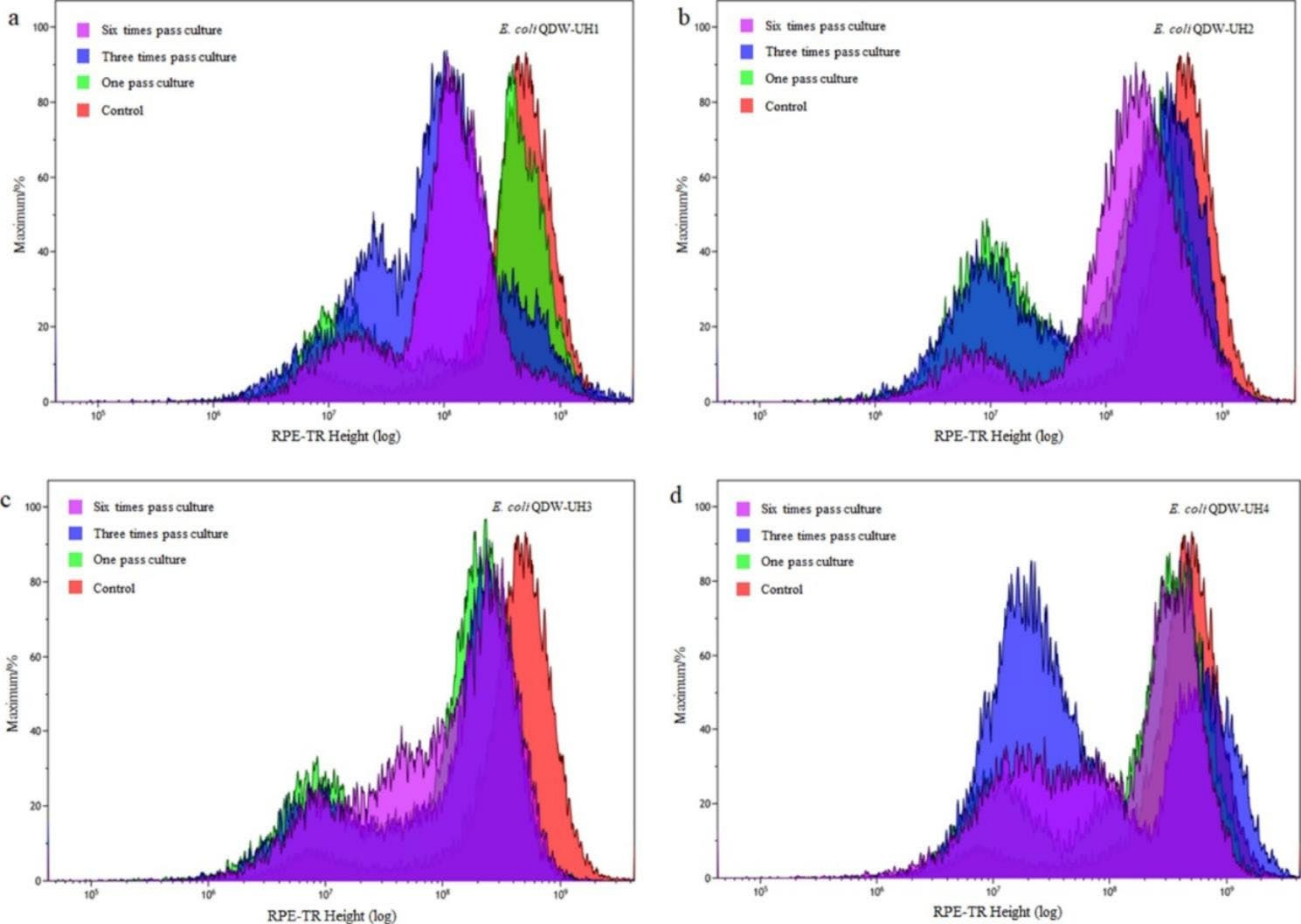
Staining fluorescence comparison between QDW and four fusion strains after passage

The prepared fusion strain was cultured in a 96-well plate at 37 ℃ for 24 h. The cells were collected and analyzed via flow cytometry. Four fusion strains, namely *E. coli* QDW-UH1, QDW-UH2, QDW-UH3, and QDW-UH4, with obvious growth advantages were selected for subsequent experimental studies. The forward scatter (FSC) and side scatter (SSC) values of QDW, QDW-UH1, QDW-UH2, QDW-UH3, and QDW-UH4 are shown in Fig. 4 (FSC-RPE-TR plots see Supplementary Fig. 1, Additional File 1). The FSC value of the original strain QDW was approximately 8.0 × 10^6^ and that of the fusion strain was approximately 2.0 × 10^7^ (Fig. 5a). Therefore, it can be concluded that the diameter of the fusion strain is significantly larger than that of the original strain QDW. The SSC value of the original strain QDW was approximately 1.0 × 10^7^, while that of the fusion strain was approximately 2.0 × 10^7^ (Fig. 5b). The genome of the fusion strain was significantly larger than that of the original QDW (Fig. 5c-e). Four fusion strains QDW-UH1, QDW-UH2, QDW-UH3, and QDW-UH4 were isolated via flow cytometry in the previous step and then subcultured. Subsequently, the bacteria were analyzed by flow cytometry, and *E.coli* QDW without mutagenesis was used as the negative control (FSC-RPE-TR plots see Supplementary Figs. 2–5, Additional File 1; FSC-SSC plots see Supplementary Figs. 6–9, Additional File 1). Comparative statistics showed that the four strains were differentiated and grouped during the first, third, and sixth generations (Fig. 6), all of which could ensure relatively stable growth, among which QDW-UH3 had the best stability.

### Stability analysis of fusion-engineered strains

**Fig. 7:**
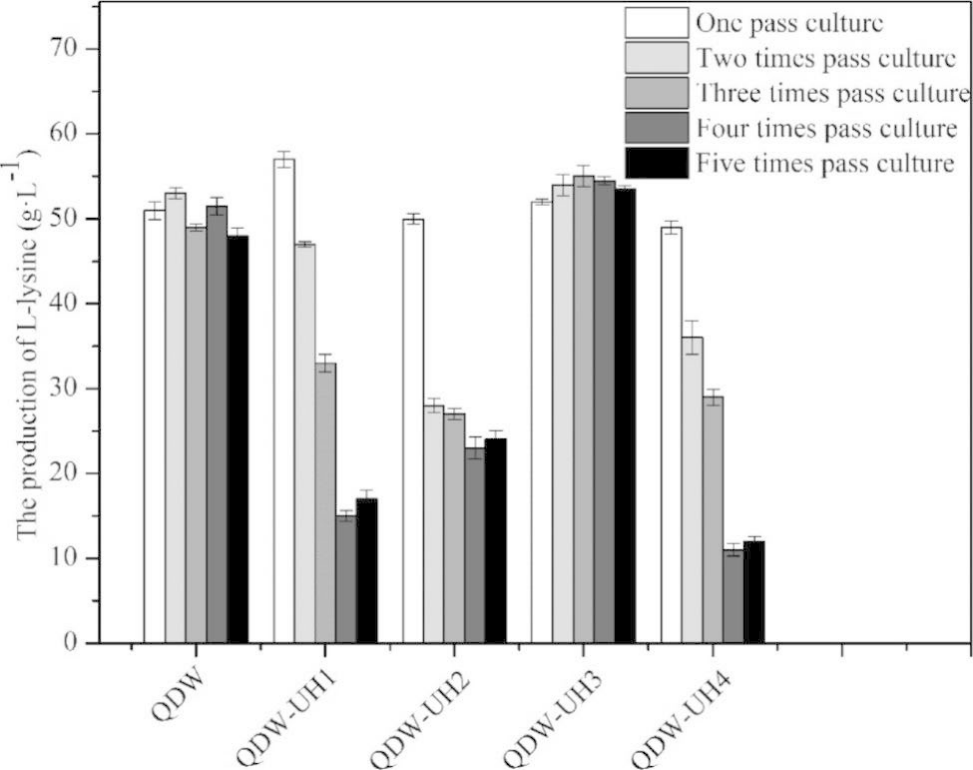
L-lysine production during five passages in QDW and four fusion-engineered strains

The fusion strains with high growth rates and high yield were screened using 96-well plates. Four of the strains, namely QDW-UH1, QDW-UH2, QDW-UH3, and QDW-UH4, were passaged five consecutive times, and the L-lysine yield was measured five times to determine whether the yield of the mutagenic strain was stable. The changes in fermentation yield of these four strains during different generations were analyzed (Fig. 7). The average L-lysine production of the original strain QDW was approximately 50.2 g/L, and that of the fusion strain QDW-UH3 was approximately 53.8 g/L, which was increased by 7.2%. The average L-lysine productivity of the original strain QDW was about 1.31 g/L/h, and that of the fusion strain QDW-UH3 was about 1.48 g/L/h (productivity changes see Supplementary Fig. 10, Additional File 1). Thus, QDW-UH3 with stable L-lysine production was obtained.

## Discussion

In the experiment of protoplast preparation conditions in this study, the formation rate and regeneration rate of protoplast preparation and recovery were used as the evaluation criteria for the optimum conditions of protoplast preparation, and the optimum bacterial age, lysozyme concentration, enzymolysis time, thermal inactivation time and ultraviolet mutagenesis time were optimized. The same lysozyme concentration was used to break the wall of QDW, and the treatment time to verify the protoplast formation rate and regeneration rate differed. The longer the lysozyme enzymolysis time, the higher the protoplast formation rate, but the lower the protoplast regeneration rate (Fig. 1b). The QDW was ruptured by enzymolysis with different concentrations of lysozyme and treated simultaneously to verify the rate of protoplast formation and regeneration. The longer the treatment time or the higher the concentration of lysozyme, the greater the impact on regeneration (Fig. 1c). The final enzymolysis time was 4 h, and the lysozyme concentration was estimated to be 0.8 mg/mL.

UV inactivation mainly occurs as a result of lethal changes in the DNA of the bacteria, meaning the protoplasts cannot regenerate alone. High-temperature thermal inactivation primarily inactivates key proteases in the somatic cytoplasm of the bacteria and also means the protoplasts cannot regenerate independently; however, thermal inactivation did not cause fatal damage at the genomic level, and involving it in fusion could partially mitigate UV-induced damage. Finally, the fusion strains with high mutation rates can regenerate through the complementary fusion of the protoplasts. In this experiment, it was found that the duration of inactivation of protoplasts played a crucial role in the subsequent protoplast fusion rate. A short inactivation time will lead to the regeneration of non-inactivated protoplasts, increasing the difficulty of fusion screening. On the other hand, if the inactivation time is too long, it may cause irreparable damage to the protoplasts of the parent strains and permanently inactivate them, leading to a decrease in the fusion rate. Studies have shown that if the time and intensity of inactivation treatment are controlled, they cannot be regenerated separately, but the proteins of UV inactivated strains are not denatured and deactivated, and the DNA of heat inactivated strains is not affected, and can be fused and regenerated according to the principle of lethal damage complementarity. The best time for UV mutagenesis of protoplasts was 7 min, and the best time for thermal inactivation of protoplasts at 85 ℃ was 30 min.

Four fusion strains, namely *E. coli* QDW-UH1, QDW-UH2, QDW-UH3, and QDW-UH4, with obvious growth advantages were selected for subsequent experimental studies. Flow cytometry can compare the relative size of cells, and the FSC corresponds to the relative size of cells. Therefore, the larger the FSC value, the larger the cell. Flow cytometry can also be used to analyze intracellular complexity. SSC corresponds to the intracellular complexity; the larger the SSC value, the more particles in the cell. Compared with those of QDW, the contents of the fusion strains were significantly higher. Furthermore, the genome of the mutant fusion strain QDW-UH3 was larger than that of QDW (Fig. 5c-e). The fusion strain showed differentiation, this may have occurred due to protoplast fusion; the genome carried by the fusion strain might have been too large, causing the strain to carry out selective discarding of genes, eventually leading to the differentiation and grouping phenomenon of the fusion strain. Among these strains, fusion strains QDW-UH1, QDW-UH2 and QDW-UH4 differentiated significantly under the premise of relatively stable growth, which was analyzed to be due to the unstable fusion progeny in the process of passage due to the fusion and exchange of genome reintegration after cell fusion. The mutagenic fusion strain QDW-UH3 (Fig. 6c) showed the most concentrated cell aggregation and the most stable growth after multiple passages.

**Fig. 8:**
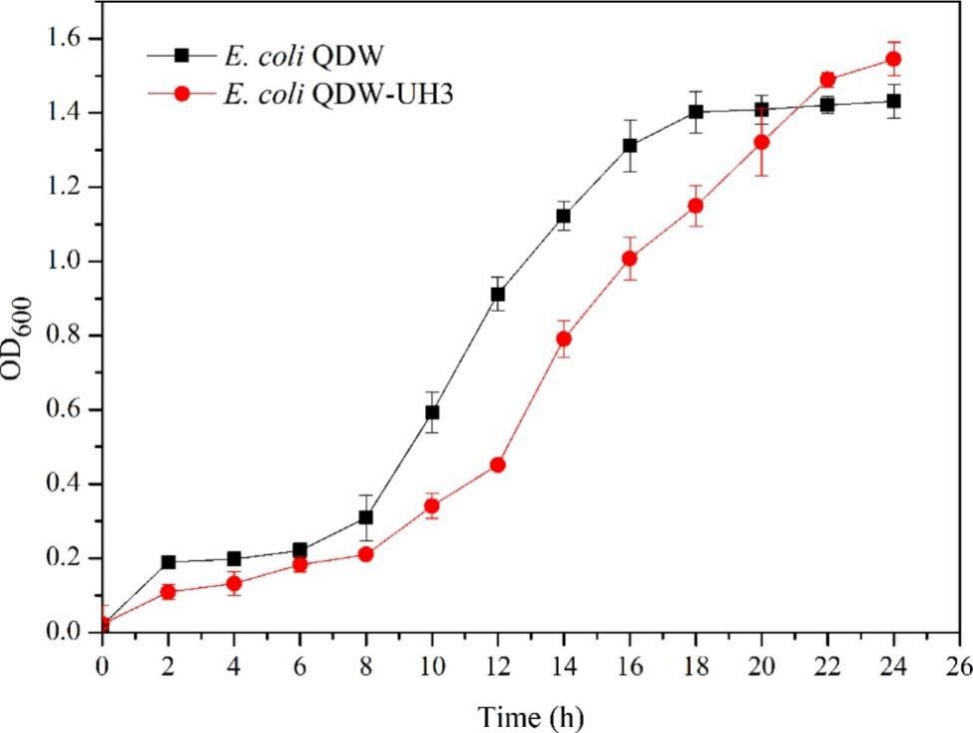
The growth curves of the original strain QDW and the fusion strain QDW-UH3

The four abovementioned fusion strains were passaged once, three times, and six times. The strains of different generations were analyzed by flow cytometry, and the growth curves of QDW-UH3 with stable grouping changes and fermentation yield were determined (Figs. 6 and 7). The average L-lysine production of the fusion strain QDW-UH3 was approximately 53.8 g/L, compared to QDW, which was increased by 7.2%. The growth rate of the fusion strain QDW-UH3 was lower than that of QDW, which might be caused by the larger genome of the fusion strain and the longer time required for replication and division (Fig. 8).

The number of amino acid transporters and mutations of the above engineered strain *E.coli* QDW-UH3 was measured and studied. It was found that *E.coli* QDW-UH3 had more stable L-lysine synthesis potential than the original strain QDW. In this study, the fusion strategy was used to improve the protoplast regeneration rate of excessive lethal ultraviolet mutations, and then the cumulative advantages were increased to improve the efficiency of *E.coli* fermentation to produce L-lysine. Thus, it is inferred that excessive UV mutagenesis causes more mutations, resulting in base mutation, deletion, or genome rearrangement. However, such mutagenesis alone can cause irreversible lethal effect on cells. We adopted the fusion strategy to improve the regeneration rate of these cells and promote the fermentation intensity of related engineering strains.

## Conclusions

In this study, protoplasts were prepared, and the formation and regeneration rate of protoplast preparation and recovery were used as the evaluation criteria for the optimal protoplast preparation conditions. The optimal bacterial age, lysozyme concentration, enzymatic hydrolysis time, heat inactivation time, and ultraviolet mutagenesis time were identified. The regenerated fusion strains were analyzed and screened via flow cytometry, followed by stability analysis and fermentation experiments. The optimal preparation time for L-lysine-producing *E. coli* QDW protoplasts was during 14–16 h of strain growth, the best concentration for enzymatic hydrolysis was 0.8 mg/mL, and the best time of enzymatic hydrolysis was 4 h. The optimal time for UV mutagenesis treatment of QDW was 7 min, and the optimal time for heat inactivation of protoplasts at 85 ℃ was 30 min. Flow cytometry analysis showed that the cell size, cell density, and genome size of the fusion strain were larger than those of QDW. Flow cytometry was used to analyze the genome quantity of fusion strains during subculture. Stable growth of *E. coli* QDW-UH1, *E. coli* QDW-UH2, *E. coli* QDW-UH3, and *E. coli* QDW-UH4 was obtained. The L-lysine yield of the fusion strain QDW-UH3 was 7.2% higher than that of QDW, indicating that the combination of the protoplast fusion technology and ultraviolet mutation breeding technology produced a fusion strain with high L-lysine yield.

Therefore, we conclude that excessive UV mutagenesis leads to the accumulation of mutations and the acquisition of more positive mutations. In order to repair the damage, reduce the lethality associated with the mutations, and improve the regeneration rate of protoplasts, homologous cell fusion was used to obtain a stable growth engineering strain. In addition, this process can promote the expression of key enzymes and increase the accumulation of target products. This efficient and concentrated dominant mutation method provides a novel approach for the targeted construction of microbial cell factories. This contributes to the fusion interaction of a variety of excellent mutant strains and improves the accumulation of target products.

## Methods

### Preparation of protoplasts

L-lysine-producing *E. coli* QDW, stored in glycerol, was streaked on an LB solid medium plate and cultured at 37 °C overnight in a constant temperature incubator. Single colonies were picked and transferred to an LB liquid medium in erlenmeyer flasks. The mixture was shaken at 37 °C for 24 h, and the bacterial concentration was recorded every 2 h. Finally, the growth curve of L-lysine-producing QDW was evaluated. In the middle and late period of the logarithmic growth phase of the bacteria, 10 mL of bacterial solution was taken and centrifuged at 2683.2 (× g) for 10 min at 4 °C. The bacteria were collected, and the SMM protoplast stable solution (pH = 6.5, 0.5 mol/L sucrose, 20 mmol/L anhydrous magnesium chloride, 0.02 mol/L maleic acid) was taken and centrifuged twice. The bacteria were suspended in 10 mL of SMM protoplast stable solution. The lysozyme solution was added and mixed well. The cells were incubated in a water bath at 37 °C, and the wall was broken by enzymatic hydrolysis. The separation process of protoplasts at 37 °C was observed under a microscope oil lens (E200MV, Nikon., Nanjing, China). Finally, the bacterial solution after enzymatic hydrolysis was centrifuged at 1509.3 (× g) for 15 min and washed once with SMM buffer; the protoplasts were then collected. The prepared protoplasts were stored in a refrigerator at 4 °C.

### Protoplasmic preparation: selection for optimal bacterial age

Our original QDW strain was purified by plate streaking and cultured in shake flasks. The OD_600_ value was measured every 2 h using an ultraviolet spectrophotometer (UV-6100, METASH, Shanghai, China) to obtain the growth curve of QDW within 24 h. The optimum time for preparing protoplasts of the strain was obtained by evaluating the growth curve of the strain.

### Protoplasmic preparation: selection of optimal enzymatic hydrolysis time

The QDW strain was activated and cultured to the mid and late logarithmic growth phases. The bacterial solution was divided into two groups, and the bacterial solution with OD_600_ = 0.8 was diluted 10 times. The lysozyme with the determined concentration was configured under the same enzymatic hydrolysis concentration (1.0 mg/mL) and set to seven time gradients of 1, 2, 3, 4, 5, 6, and 7 h for the enzymatic hydrolysis treatment. At the same time, a control group without lysozyme treatment was set up to calculate the formation rate, verify the effect of enzymatic hydrolysis time on the formation of protoplasts of parental strains, and obtain the optimal enzymatic hydrolysis time by measurement. Immediately after completing enzymatic digestion, the bacteria were centrifuged twice (4 °C, 1509.3 (× g), 10 min). The bacterial solution was diluted 100 times with SMM buffer solution, and 0.1 mL of bacterial solution was taken after allowing it to stand for 30 min. The bacterial solution was evenly coated on the LB regeneration medium and cultured in an incubator at 37 °C for 24 h. On the second day, the regenerated colonies on the plate were observed, and the regeneration rate was calculated to measure the optimal enzymatic hydrolysis time. Protoplast formation rate and regeneration rate were calculated (Eqs. 1 and 2).

1 $${\text{Protoplast formation rate}} = \left( {A - B} \right)/{\text{A}} \times 100\%$$

2 $${\text{Regeneration rate}} = \left( {C - B} \right)/\left( {A - B} \right) \times 100\%$$

where A is the number of colonies on the LB regeneration plate before lysozyme treatment; B is the number of colonies that did not become protoplasts after lysozyme treatment; C is the number of colonies on LB regeneration plates after lysozyme treatment.

### Protoplasmic preparation: selection of optimal enzyme treatment concentration

The QDW was activated and cultured to the mid and late logarithmic growth phases. The bacterial solution was divided into two groups, and the bacterial solution with OD_600_ = 0.8 was diluted 10 times. Six different concentrations of lysozyme solution were prepared, namely 0.2, 0.4, 0.6, 0.8, 1.0, and 1.2 mg/mL lysozyme solution, and a control group without lysozyme treatment was set up to calculate the formation rate. The bacterial solution was diluted 10 times with SMM buffer solution, and a small amount of bacterial solution was taken and evenly coated on the LB regeneration medium. Enzymolysis was carried out at 30 °C, under different concentrations in a gradient. Immediately after enzymolysis, the cells were centrifuged (1509.3 (× g), 10 min) and washed twice with buffer. The bacterial solution was diluted 100 times with SMM buffer solution, and 0.1 mL of bacterial solution was taken after allowing it to stand for 30 min and coated on LB medium. The number of regenerated colonies on the plate was observed after overnight incubation in a constant temperature incubator at 37 °C, and the regeneration rate was calculated to measure the optimal enzymatic hydrolysis concentration.

### Inactivated protoplast UV mutagenesis and heat treatment

The mutant strains were obtained by excessive ultraviolet mutagenesis, and the regenerated complementary fusion strains were obtained by combining this with heat inactivation. This method can increase the mutation rate and also be used as a marker screening condition for the screening of fusion strains.

The process to prepare the inactivation marker of protoplasts was divided into two steps. The first step was ultraviolet inactivation. The prepared protoplast culture medium was evenly coated on a disposable plate, and ultraviolet mutagenesis was performed at a distance of 40 cm from the ultraviolet lamp. Nine time periods of 0, 1, 2, 3, 4, 5, 6, 7, and 8 min were set up, and the protoplasts were subjected to UV mutagenesis under a 15 W UV lamp on a clean bench. After mutation, attention was paid to avoid light treatment, avoiding light repair of errors caused by UV mutation treatment. The protoplasts that had undergone UV mutagenesis were grouped and treated. One group was measured for the lethal curve of UV mutagenesis. The UV-treated protoplasts exposed for different time periods were uniformly coated on a sterile LB regeneration plate and transferred to a 37 °C constant temperature incubator for cultivation. The other group was named UV-lys and placed at 4 °C for preservation and preparation for the next protoplast fusion.

In the second step, the parental strains were exposed to high-temperature inactivation treatment. The prepared protoplast culture solution was distributed into 1.5 mL sterilized centrifuge tubes and placed in an 85 °C water bath for heat treatment. Eight time gradients of 5, 10, 15, 20, 25, 30, 35, and 40 min were set. The protoplasts treated at 85 °C were grouped. One group of protoplasts treated in a hot water bath for different times was coated, and the lethal curve of heat-inactivated protoplasts was measured. The other group was named H-lys and stored at 4 °C to prepare for the next protoplast fusion.

### Fusion of protoplasts

The UV-lys and H-lys protoplasts were mixed at a volume of 0.5 mL each and centrifuged at 1509.3 (× g) for 10 min at 4 °C. Subsequently, the protoplast precipitation was collected, and 4.8 mL of 40% PEG6000 solution and 0.2 mL of new calcium phosphate solution were added and gently mixed [39]. The mixture was incubated at 30 °C for 15–30 min. The fusion process was observed under a microscope, and the morphology of strains at each stage of the protoplast preparation process was recorded using an optical microscope. After centrifugation at 1048.1 (× g) for 10 min at 4 °C and washing with SMM buffer once, 0.5 mL SMM buffer was resuspended, and 100 µL was coated on LB solid medium plate. The plate was cultured in a 37 °C incubator for 2 days.

### Screening of fusion strains

Flow cytometry mainly consists of a flow chamber and fluid flow system, a light source and optical system, a signal collection and conversion system, and a computer and analysis system [40, 41]. The strength of forward scatter (FSC) reflects the measured cell’s size, and the strength of side scatter (SSC) reflects the complexity of intracellular particles, mainly used to obtain relevant information about the fine structure inside cells. The fusion strains with larger volumes and higher contents were selected by flow cytometry. Fluorescence intensity is the response value of the cell or the fluorescent dye on the cell after being excited by the laser. The positive cells with different fluorescence intensities are sorted.

After mutagenesis and inactivation, the fusion strains were sorted by flow cytometry, and the strains with different volume sizes and inclusion contents were screened. We used 96-well plates to screen the strains with a higher growth rate, which could improve the efficiency of screening strains. Regeneration medium (200 µL) was added to each well, and each strain was punched into different wells by flow cytometry. The plate was left to stand for about 5 min and then placed into an incubator at 37 °C. Subsequently, the concentration of bacteria was measured using a microplate reader. The cell culture plate was lifted to observe the light from the bottom up to see whether the cells were gathered. The plate was then oscillated slightly using a flat oscillator. Cells should be as dispersed as possible so that most cells are in a single state. The yield of L-lysine was measured by an SBA biosensor analyzer (BSA-90, Jinan Yanke Co., Ltd., Jinan, China) after 48 h of culture. Ten fusion strains with higher growth rates were selected, and four strains with higher yields were selected through one-generation fermentation.

### Stability analysis of the fusion strain

After the four abovementioned fusion strains were activated, a shake flask fermentation test was carried out. Fermentation medium, pH = 7.2, glucose 20 g/L, corn pulp 1 g/L, ammonium sulfate 10 g/L, magnesium sulfate 2 g/L, potassium chloride 1 g/L, copper sulfate 10 mg/L, zinc sulfate 10 mg/L, ferrous sulfate 50 mg/L, manganese sulfate 50 mg/L. The bacteria were inoculated into 50 mL medium and cultured at 37 °C and 220 r/min. Each strain was passaged five times, and each generation was fermented for 48 h. Each strain’s growth curve and L-lysine production were measured, and the OD_600_ value was measured every 2 h to draw the cell growth curve. In addition, sampling was carried out every 12 h, and the content of L-lysine was measured using an SBA biosensing analyzer. Finally, the mutant fusion strains with stable yield were screened out.

## Electronic supplementary material

Below is the link to the electronic supplementary material.

Supplementary Material 1

## Data Availability

The dataset supporting the conclusions of this article is included within the article and its additional file.

## Abbreviations

EDTA: Ethylene Diamine Tetraacetic Acid
MNNG: N-methyl-N′-nitro-N-nitrosoguanidine
AEC: S-2-aminoethyl-L-cysteine
UV: Ultraviolet
LB: Luria-Bertani
FSC: Forward scatter
SSC: Side scatter
