# Supplementary material for: Improving the regeneration rate of deep lethal mutant protoplasts by fusion to promote efficient L-lysine fermentation
Source: BMC Biotechnol. 2023 Jul 14;23:22. doi: 10.1186/s12896-023-00792-8 (PMC10347866; doi:10.1186/s12896-023-00792-8)
Supplement: Supplementary file 1 — Supplementary Material 1 [file 12896_2023_792_MOESM1_ESM.docx]

Supplementary Figure 1


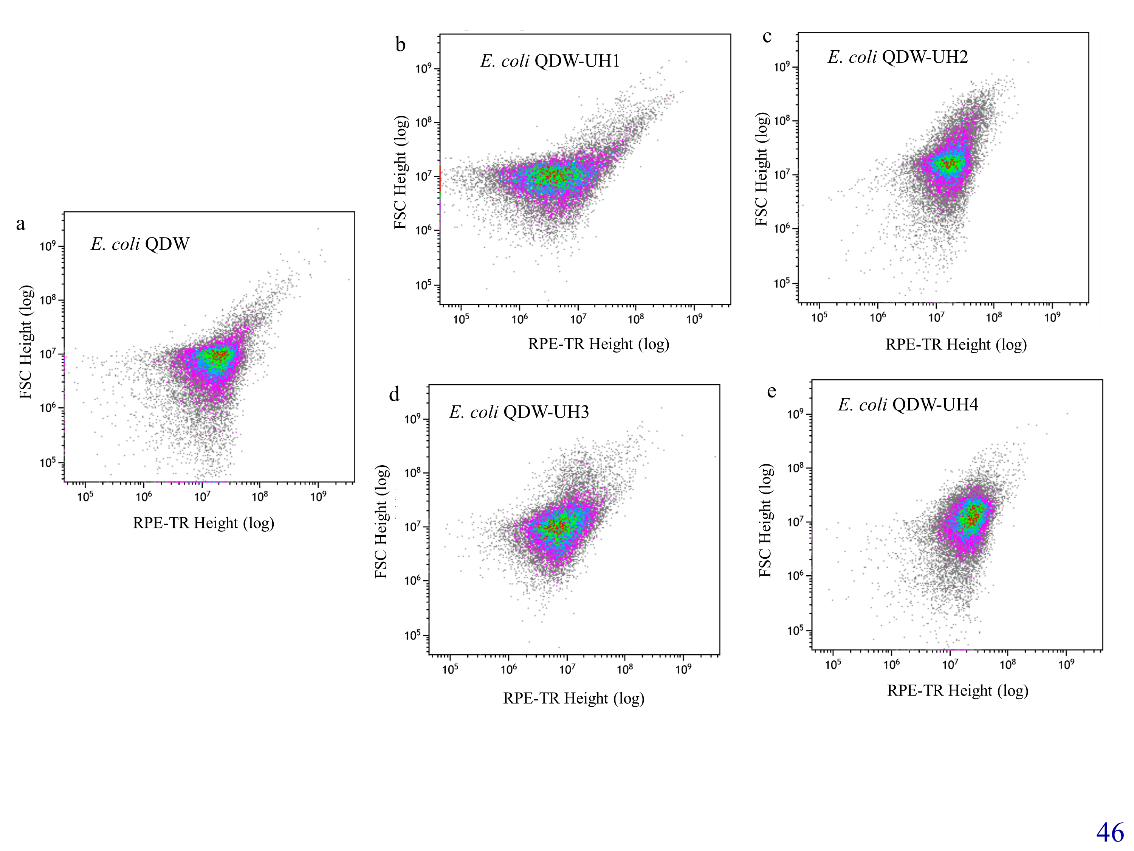


Figure. 1 FSC-RPE-TR plots of QDW and fusion strains by flow cytometry

Supplementary Figure 2


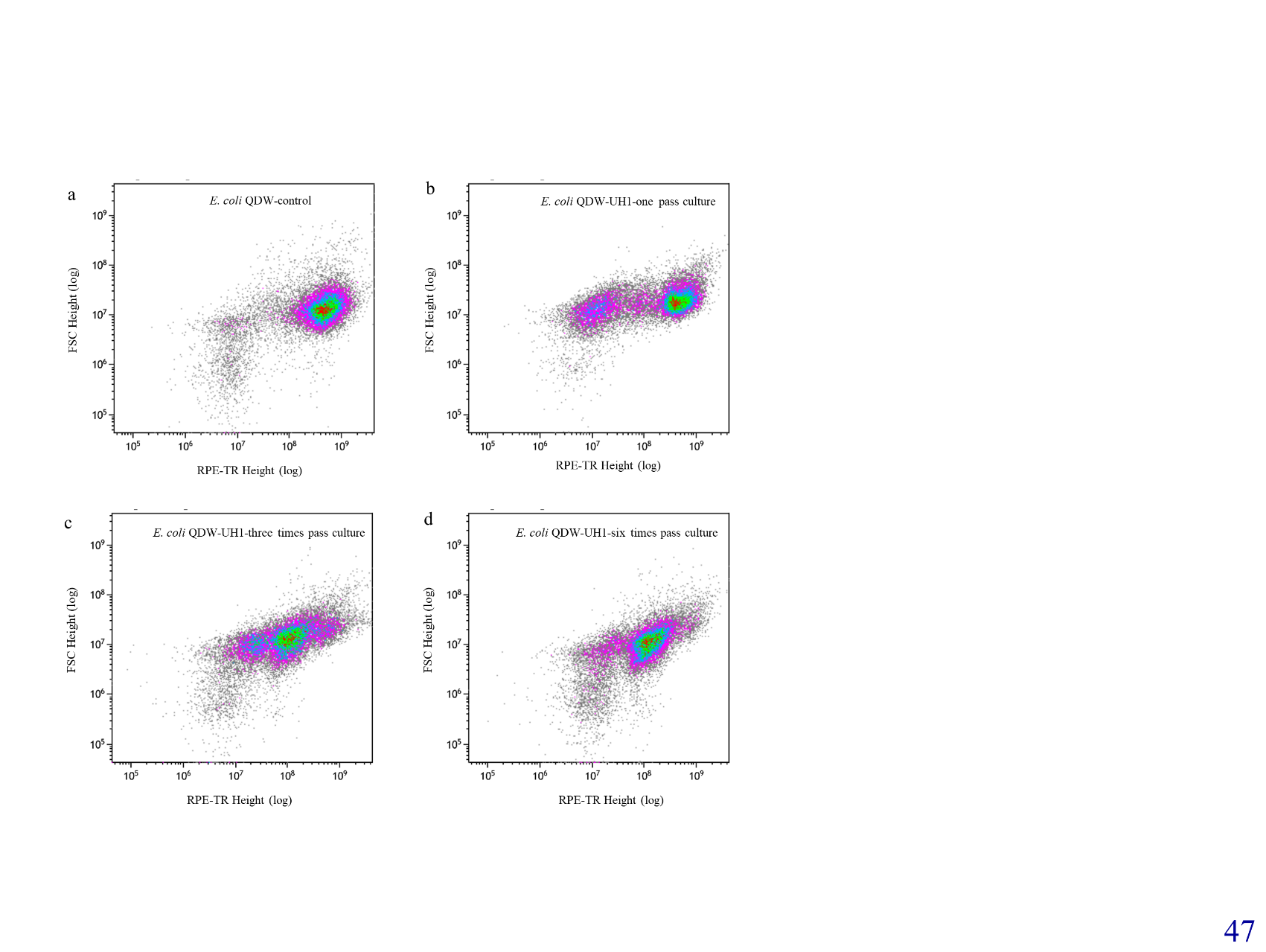


Figure. 2 FSC-RPE-TR plots of fusion strains QDW-UH1 by flow cytometry

Supplementary Figure 3


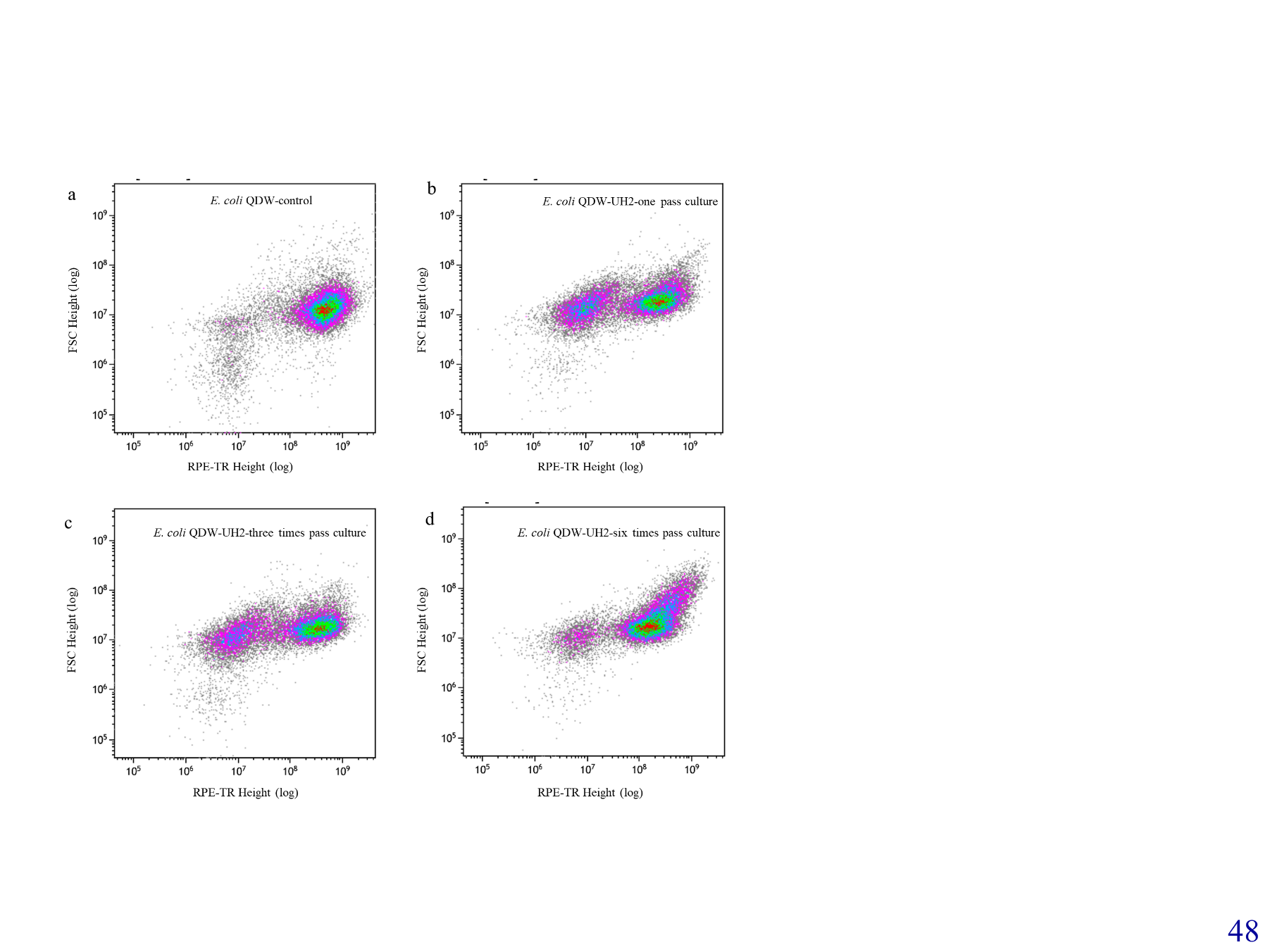


Figure. 3 FSC-RPE-TR plots of fusion strains QDW-UH2 by flow cytometry

Supplementary Figure 4


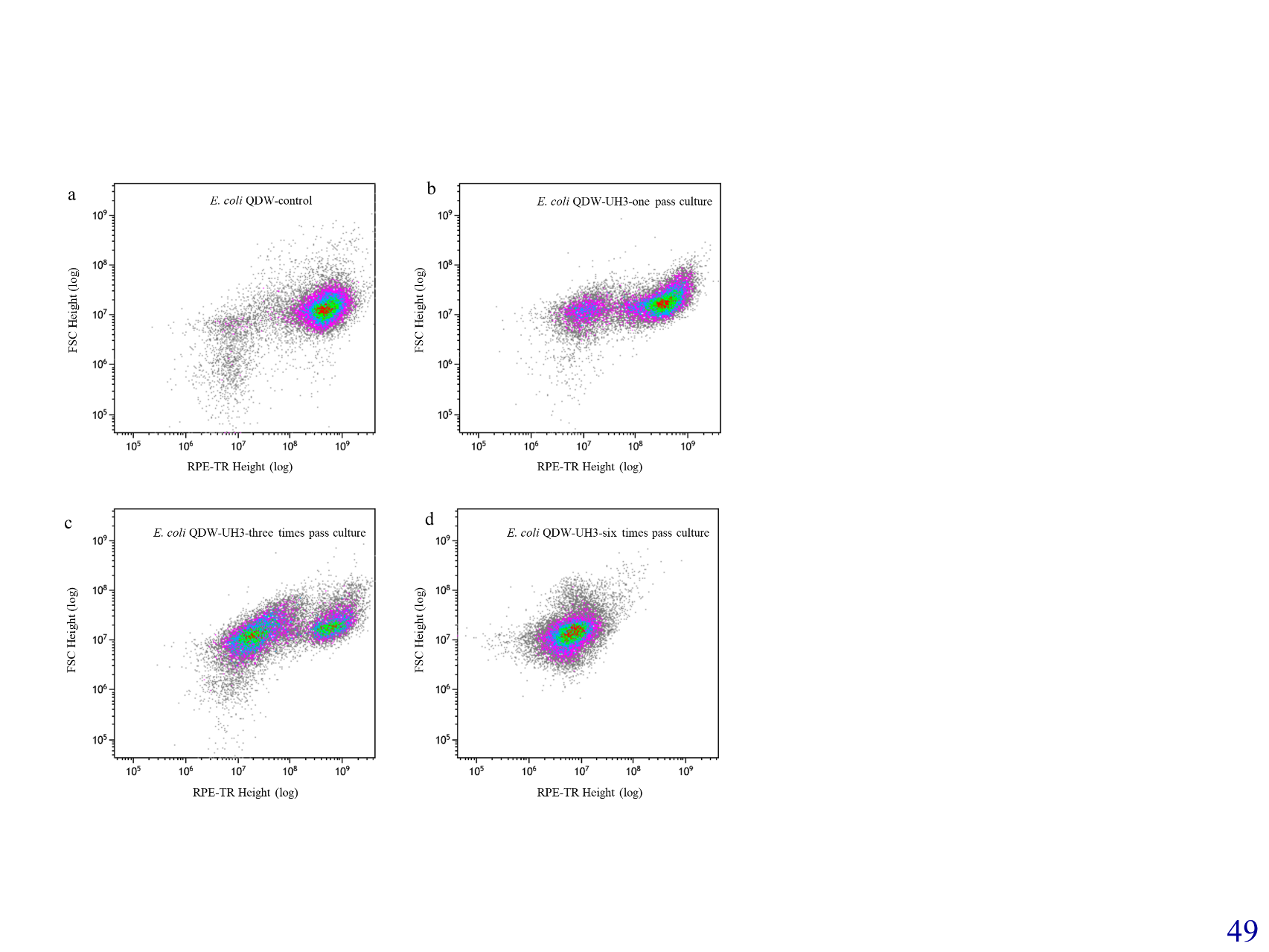


Figure. 4 FSC-RPE-TR plots of fusion strains QDW-UH3 by flow cytometry

Supplementary Figure 5


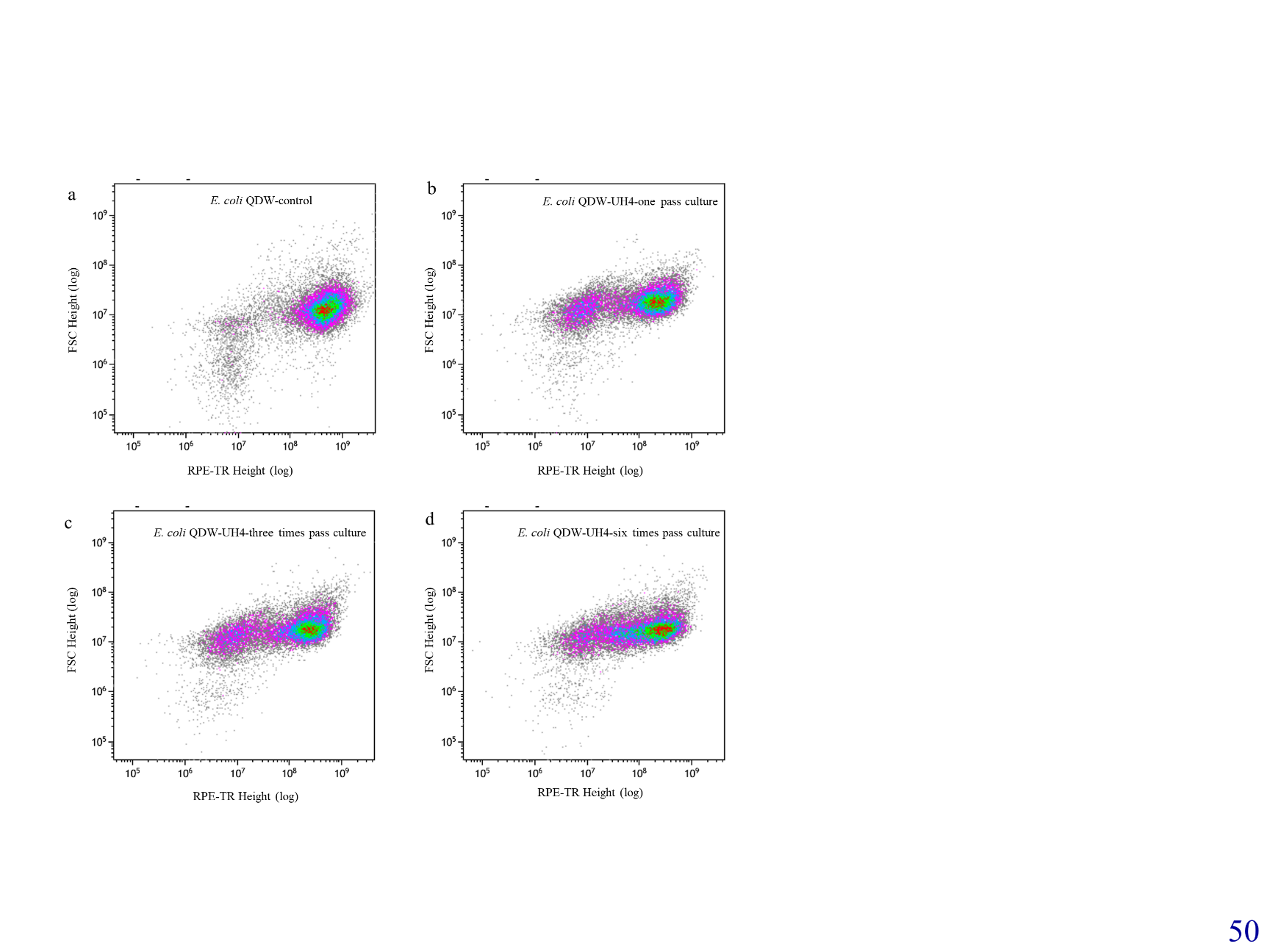


Figure. 5 FSC-RPE-TR plots of fusion strains QDW-UH4 by flow cytometry

Supplementary Figure 6


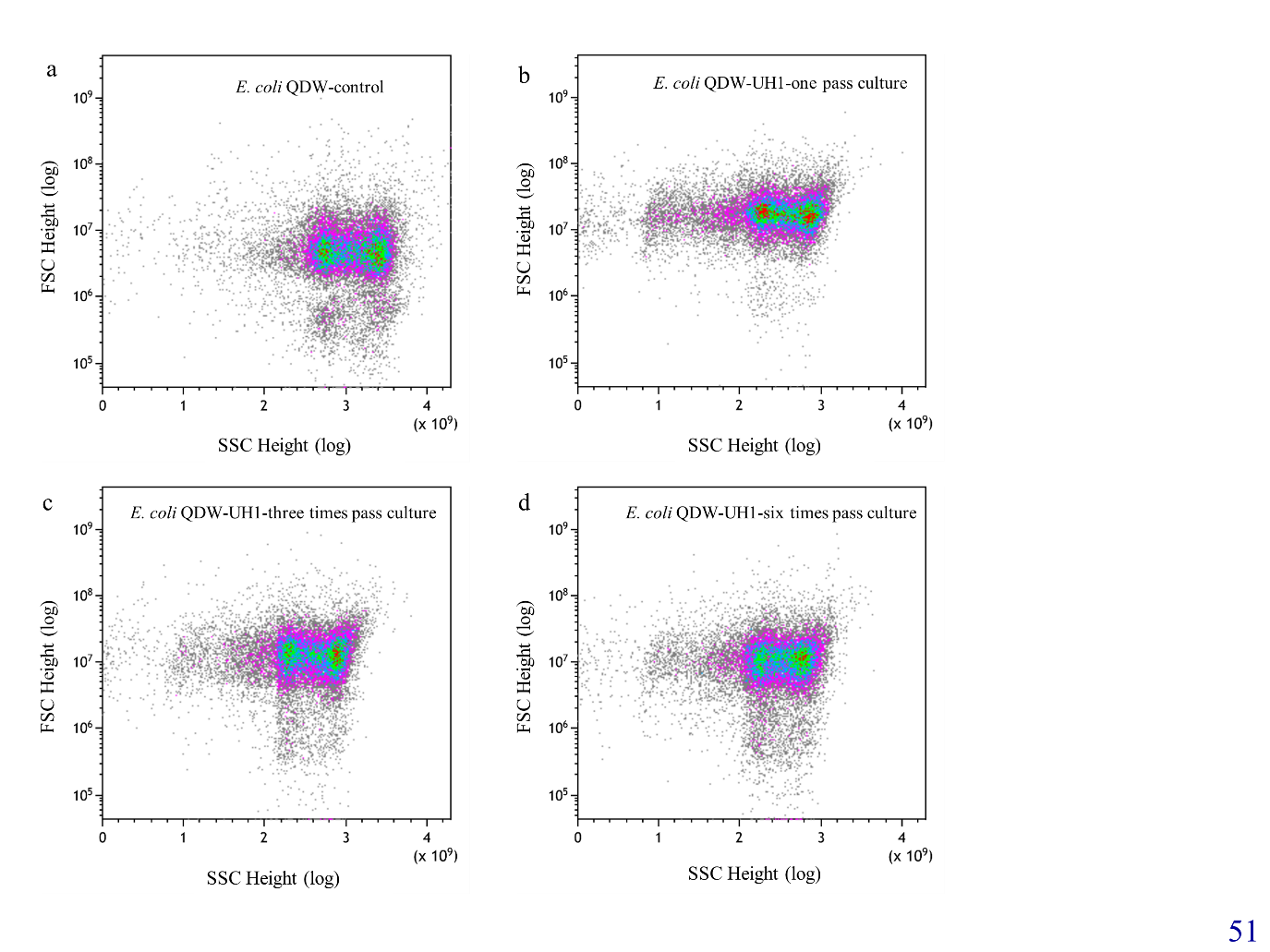


Figure. 6 FSC-SSC plots of fusion strains QDW-UH1 by flow cytometry

Supplementary Figure 7


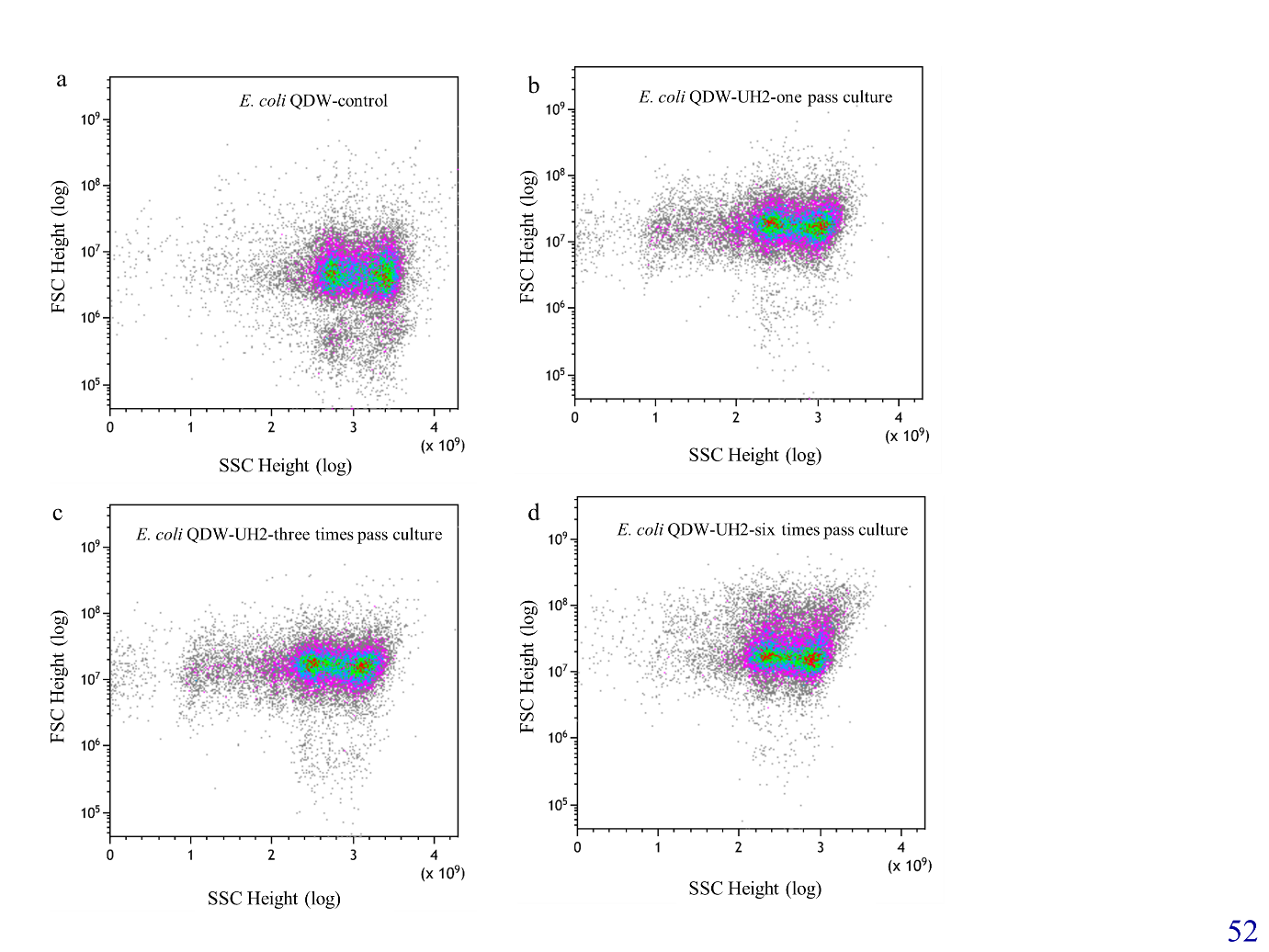


Figure. 7 FSC-SSC plots of fusion strains QDW-UH2 by flow cytometry

Supplementary Figure 8


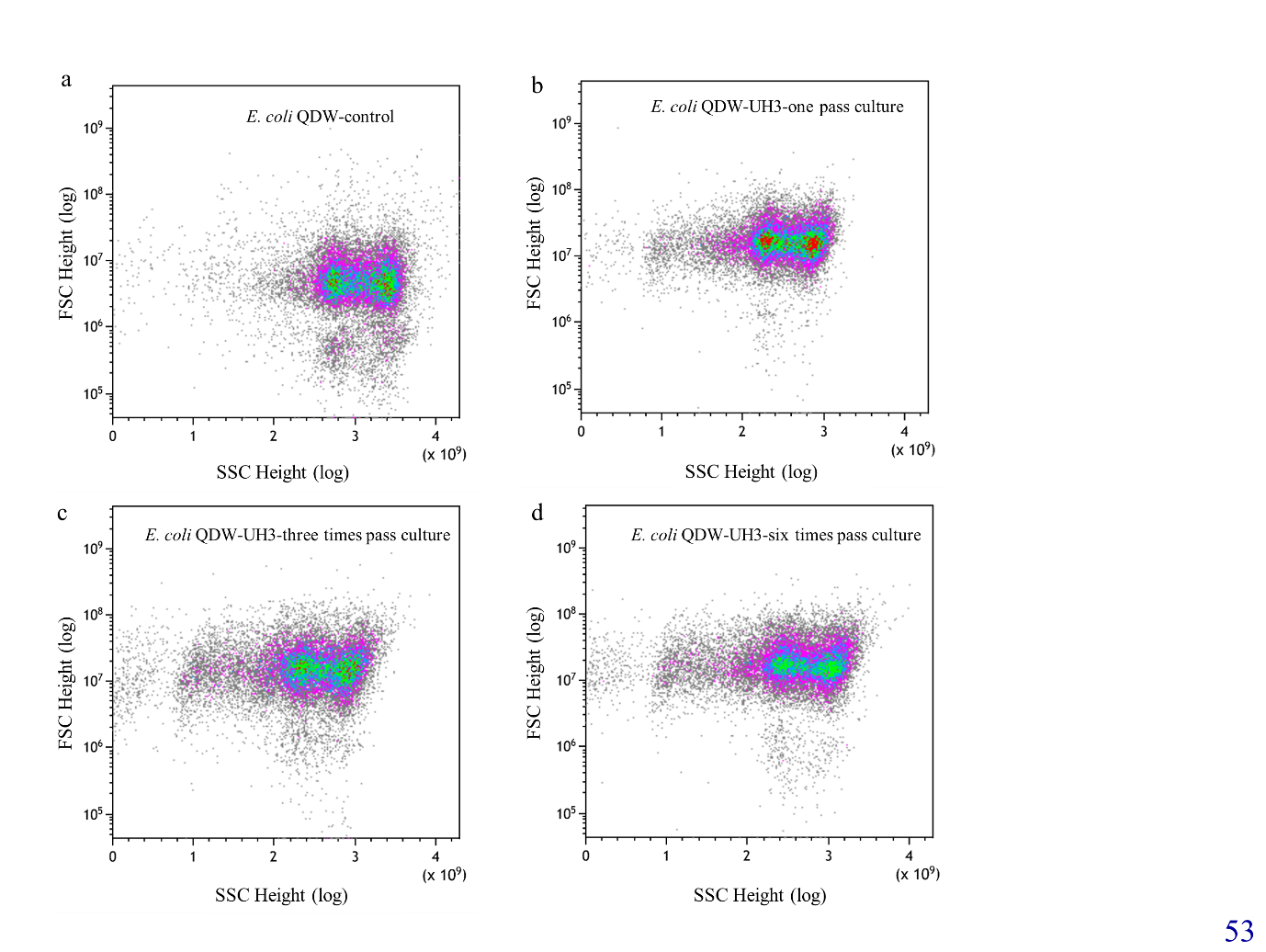


Figure. 8 FSC-SSC plots of fusion strains QDW-UH3 by flow cytometry

Supplementary Figure 9


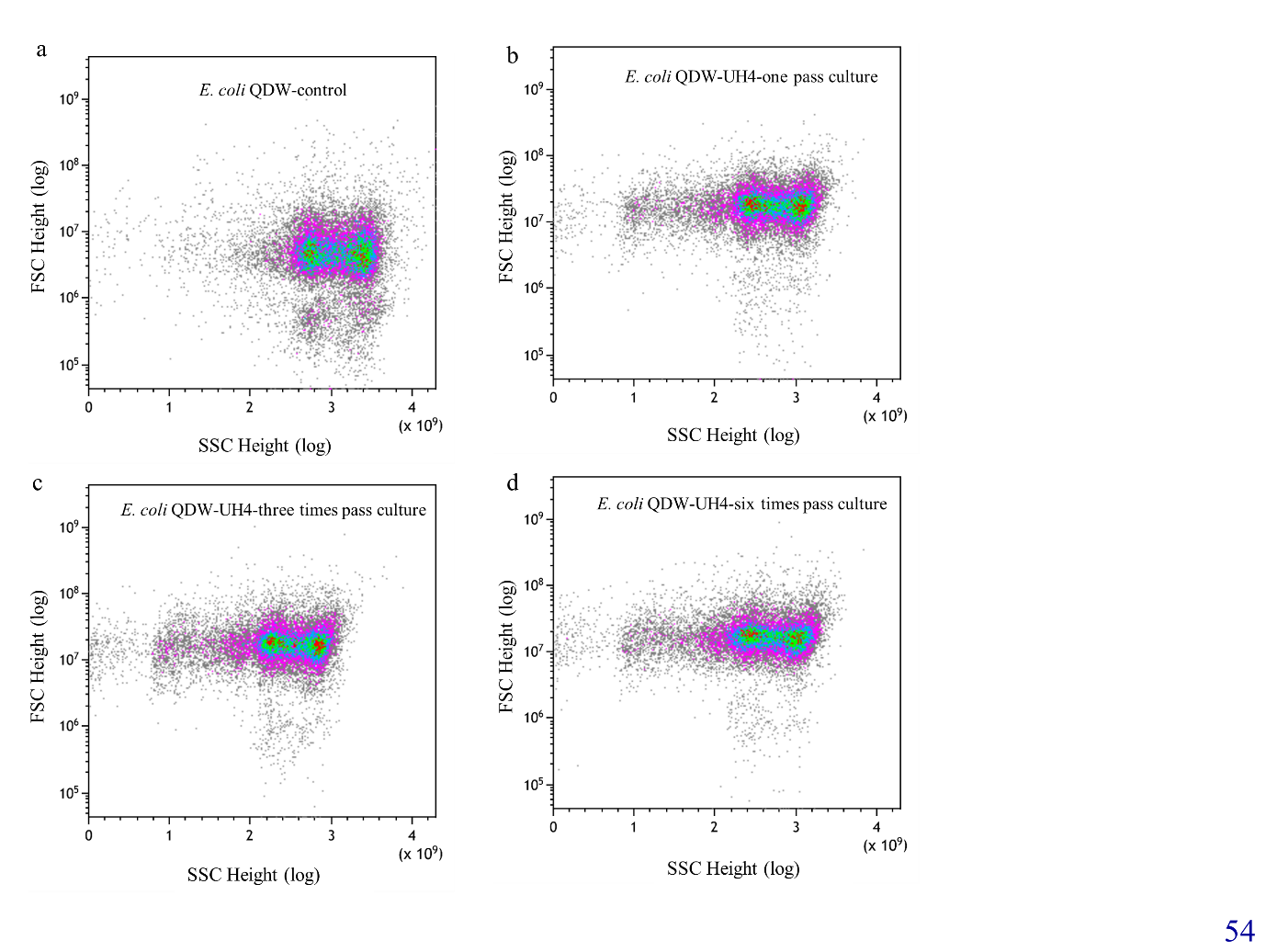


Figure. 9 FSC-SSC plots of fusion strains QDW-UH4 by flow cytometry

Supplementary Figure 10





Figure. 10 L-lysine productivity in five generations of QDW and four mutant-fusion-engineered strains
